# Unprecedented Megakaryocytic Blast Phase Transformation in Chronic Myeloid Leukemia After 16 Years of Tyrosine Kinase Inhibitor Therapy

**DOI:** 10.7759/cureus.67443

**Published:** 2024-08-21

**Authors:** Kaworu Takatsuna, Shuhei Kurosawa, Hitomi Nakayama, Aki Sakurai, Chisako Ito, Yoshinobu Aisa, Tomonori Nakazato

**Affiliations:** 1 Department of Hematology, Yokohama Municipal Citizen’s Hospital, Yokohama, JPN; 2 Division of Transfusion and Cell Therapy, Tokyo Metropolitan Cancer and Infectious Diseases Center, Tokyo, JPN

**Keywords:** hematopoietic stem cell transplant, ponatinib, tyrosine kinase inhibitor (tki), chronic myeloid leukemia (cml), megakaryocytic blast phase

## Abstract

We report the case of a 51-year-old Japanese man with chronic myeloid leukemia (CML) initially diagnosed in the chronic phase. For 16 years, the patient maintained chronic phase (CP) under treatment with first- and second-generation tyrosine kinase inhibitors (TKIs), including imatinib, dasatinib, and bosutinib, none of which resulted in ABL1 mutations. However, despite long-term disease stability, the patient experienced an abrupt progression to the megakaryocytic blast phase (MBP), a rare and aggressive form of CML. In response to this progression, ponatinib, a third-generation TKI, was introduced as a fourth-line therapy. Remarkably, within 7 months of initiating ponatinib, the patient achieved a deep molecular response (DMR), evidenced by a reduction in *BCR::ABL1* transcript levels to undetectable levels (MR^5.0^). This molecular remission enabled the patient to proceed with an allogeneic bone marrow transplantation from a human leukocyte antigen (HLA) 8/8-allele-matched unrelated donor. Post-transplantation, the patient has maintained DMR for 14 months without recurrence, despite the challenges posed by graft-versus-host disease. This case illustrates the critical role of third-generation TKIs like ponatinib in managing advanced CML phases, especially when previous therapies fail. It also emphasizes the necessity of vigilant long-term monitoring during the chronic phase to detect and address any signs of disease progression promptly.

## Introduction

The advent of tyrosine kinase inhibitors (TKIs) has significantly improved the prognosis of chronic myeloid leukemia (CML), particularly during its chronic phase (CP) [1]. Imatinib, the first-generation TKI, functions by specifically inhibiting the *BCR::ABL1* tyrosine kinase, a fusion protein resulting from the Philadelphia chromosome translocation, which drives the uncontrolled proliferation of leukemic cells. Second-generation TKIs, such as dasatinib and bosutinib, offer broader kinase inhibition, targeting not only *BCR::ABL1* but also other kinases involved in disease progression, thus providing effective treatment options in cases where resistance to imatinib has developed.

However, despite the efficacy of these therapies, CML can still progress to the blast phase (BP), which presents a significant clinical challenge and often necessitates allogeneic hematopoietic stem cell transplantation (allo-HSCT) for potential cure [2, 3]. Among BP cases, megakaryocytic blast phase (MBP) in CML (CML-MBP) is rare, and its clinical features and treatment strategies are not well defined. Here, we present a case of CML-MBP that was initially diagnosed in CP and later progressed to MBP after prolonged treatment with TKIs. The patient achieved a deep molecular response (MR) with ponatinib as the fourth-line treatment and subsequently underwent successful bone marrow transplantation (BMT).

## Case presentation

A 51-year-old Japanese man with a history of type II diabetes mellitus, hyperlipidemia, and anal fissure was diagnosed with CML-CP in 2006 at Yokohama Rosai Hospital, Japan. The patient was initially treated with imatinib but was switched to dasatinib in 2017, although the specific reasons for the change were not detailed. In May 2017, he was transferred to our hospital with prolonged, unexplained diarrhea. Suspected drug-induced diarrhea led to a switch from dasatinib to bosutinib, starting at 100 mg/day. Following the switch, the patient’s diarrhea resolved, and the dose of bosutinib was gradually increased to 300 mg/day. The treatment efficacy was assessed using the International Scale (IS) for *BCR::ABL1*, and although a major MR was achieved, MR^4.0^ was not reached. Despite this, the patient expressed a preference to continue bosutinib therapy.

**Figure 1:**
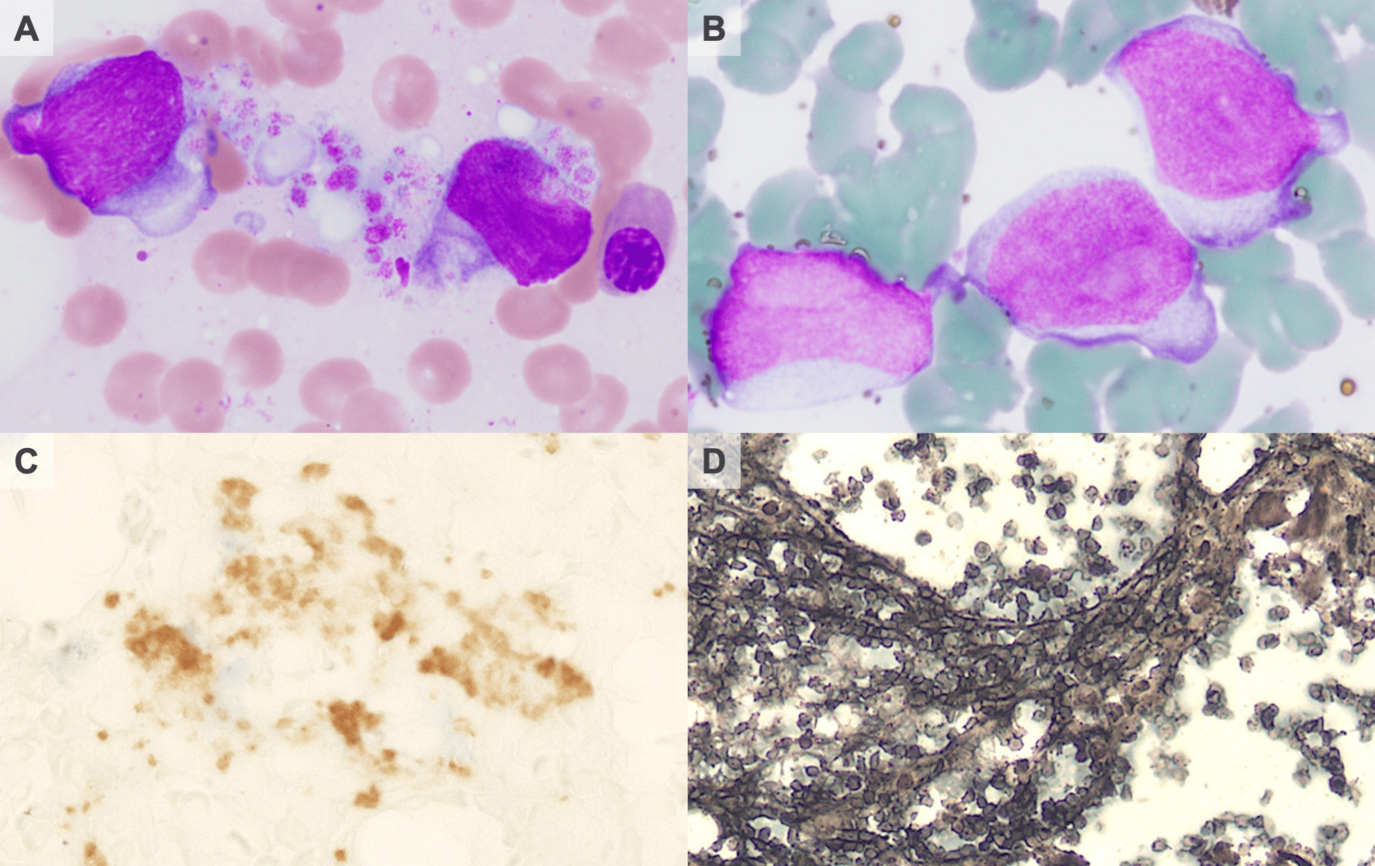
Bone marrow examination on admission A) Blasts with cytoplasmic blebs and platelet anisocytosis in May-Giemsa stain. B) Myeloperoxidase stain showing negative in megakaryoblasts. C) Immunohistochemistry with CD42b showing positive in blasts. D) Grade I fibrosis on silver impregnation stain.

On May 13th, 2022, the patient was admitted with fever. Physical examination revealed no abnormalities, but laboratory tests showed a white blood cell count of 170,060/μL (blasts 7.0%), a platelet count of 555,000/μL, hemoglobin of 14.4 g/dL, and lactate dehydrogenase of 1,510 U/L. The IS for *BCR::ABL1* in the peripheral blood was 107.2%. Owing to the dry tap, bone marrow aspiration yielded scant samples but showed a predominance of blasts with a high nucleus-to-cytoplasm (N/C) ratio and bleb-like features (Figure 1A). Myeloperoxidase staining was negative (Figure 1B). Biopsy indicated CD34- and CD42b-positive blasts with high N/C ratios and myelofibrosis (MF), graded as MF-1 (Figure 1C, D). Cytogenetic analysis revealed the presence of 46,XY,t(9;22)(q34.1, q11.2) in 20 cells. Genetic testing was positive for the major *BCR::ABL1* without *ABL1 *kinase domain (KD) mutation, confirming MBP diagnosis.

Ponatinib (45 mg/day) was initiated, but it was temporarily discontinued when the patient was admitted with complaints of abdominal pain. Laboratory tests revealed a slight increase in lipase level (87 U/L), and an abdominal CT scan showed enlargement in the pancreatic tail and surrounding retroperitoneal fat stranding, suggesting drug-induced pancreatitis. Ponatinib (30 mg/day) was resumed 12 days later with no further adverse effects. The patient achieved deep MR (IS for *BCR::ABL1*, 0.0047%) after 4 months and MR^5.0^ (IS for *BCR::ABL1*, below the detection limit) at 7 months post-initiation of ponatinib.

**Figure 2:**
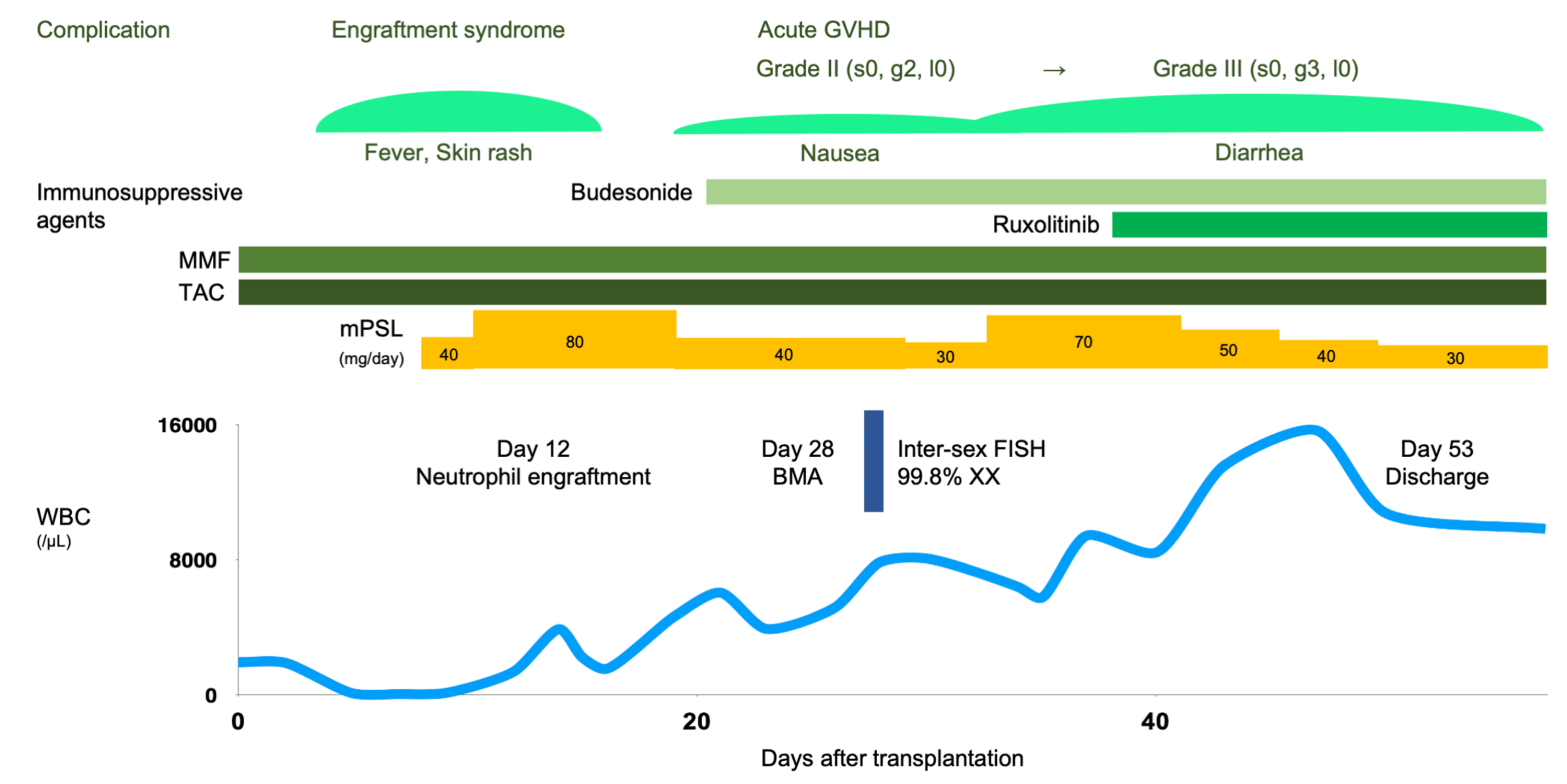
Clinical course of the patient after bone marrow transplantation BMA, bone marrow examination; FISH, fluorescence in situ hybridization; g, gut; GVHD, graft-versus-host disease; l, liver; MMF, mycophenolate mofetil; mPSL, methylprednisolone; s, skin; TAC, tacrolimus.

Allo-HSCT was planned initially; however, no related donors were available. In January 2023, the patient underwent unrelated BMT using a human leukocyte antigen (HLA) 8/8-allele-matched donor. Figure 2 illustrates the post-transplant course of the case. The conditioning regimen included fludarabine (180 mg/m2), busulfan (12.8 mg/kg), and melphalan (80 mg/m2) along with tacrolimus and mycophenolate mofetil for graft-versus-host disease (GVHD) prophylaxis. On day 12 post-BMT, neutrophil engraftment was achieved, and 1 mg/kg methylprednisolone was initiated to treat the engraftment syndrome. The patient was diagnosed with acute GVHD grade II (skin, stage 0; gut, stage 1; liver, stage 0) on day 34 and successfully managed with ruxolitinib in addition to prophylactic drugs, leading to discharge on day 52. The patient maintained MR^5.0^ 14 months post-BMT. Ponatinib was discontinued before BMT, and TKI was not administered post-transplant.

## Discussion

Table 1 summarizes 12 previously reported cases of CML-MBP [4-14]. Except for one case, all were diagnosed in BP at the initial presentation of CML. Three cases underwent allo-HSCT, and one case included maintenance therapy with ponatinib post-transplantation. Our patient was initially diagnosed in CP and transitioned to BP during prolonged TKI therapy, without additional chromosomal abnormalities or *BCR::ABL1* mutations detected via Sanger sequencing. The higher detection limit of Sanger sequencing, compared to mass spectrometry, next-generation sequencing, and droplet digital polymerase chain reaction (PCR), may have contributed to the absence of identified mutations [15]. The observed efficacy of ponatinib without intensive chemotherapy might suggest a potential resistance to first- or second-generation TKIs, even though no ABL1 Kinase Domain (KD) mutations were detected.

**Table 1:** Summary of reported cases of megakaryocytic blast phase in chronic myeloid leukemia ADR, adriamycin; AraC, cytarabine; BMT, bone marrow transplantation; CML, chronic myeloid leukemia; DNR, daunorubicin; ETP, etoposide; HU, hydroxyurea; IDR, idarubicin; MTX, methotrexate; NA, not available; PBSCT, peripheral blood stem cell transplantation; PSL, prednisolone; TKI, tyrosine kinase inhibitor; VCR, vincristine.

Reference number	Reported year	Age/Sex	Disease status at CML Dx	TKI after BP	Other chemotherapy	Subsequent HSCT	Outcomes
Hirose Y, et al. [4]	2002	42/F	Blast phase	Imatinib	VCR+PSL, MTX+6-MP	None	Alive
Pelloso LA, et al. [5]	2002	25/F	Blast phase	Imatinib	DNR+AraC	None	Alive
Bryant BJ, et al. [6]	2007	53/F	Blast phase	None	HU	None	Death
Campiotti L, et al. [7]	2007	60/F	Blast phase	Imatinib	None	None	Alive
Pullarkat ST, et al. [8]	2008	62/M	Blast phase	Imatinib	HU, IDR+AraC	None	Death
Karkuzhali P, et al. [9]	2013	36/F	Blast phase	Imatinib	ETP+ADR+AraC	None	Alive
Hino Y, et al [10]	2016	58/F	Blast phase	Imatinib	None	BMT	Alive
Khemka R, et al. [11]	2019	31/F	Blast phase	Imatinib	ETP+ADR+AraC	None	Not reported
		70/M	Blast phase	Imatinib	ETP+ADR+AraC	None	
		36/M	Chronic phase	NA	NA	NA	
Sasaki H, et al. [12]	2019	42/F	Blast phase	Ponatinib	None	BMT	Alive
Ureshino H, et al. [13]	2019	35/M	Accelerated phase	Ponatinib	AraC	PBSCT	Death
Agrawal S, et al. [14]	2022	22/M	Blast phase	Dasatinib	DNR+AraC	None	NA
The present case	2024	51/M	Chronic phase	Ponatinib	None	BMT	Alive

Prognostic factors for allo-HSCT in CML indicated poorer outcomes in BP than in CP or accelerated phase, although remission at transplantation is associated with better prognosis [2]. A registry-based study in Japan reported improved outcomes in BC with the use of second- or third-generation TKIs [3]. In this case, although the patient progressed to MBP, ponatinib use achieved MR pre-transplant, and the patient showed no signs of relapse post-transplant. However, there are reports of late relapse post-transplantation in CML, suggesting that the survival curves do not plateau as compared to those of acute leukemia [16, 17]. Post-transplant TKI therapy to prevent relapse has been evaluated, with single-arm prospective trials indicating the efficacy and safety of imatinib or nilotinib in relapse prevention [18-20]. According to the latest National Comprehensive Cancer Network (NCCN) guidelines, TKI therapy for at least one year should be considered in patients with prior BP, even if *BCR::ABL* quantitative polymerase chain reaction (qPCR) is negative [1]. The present case was complicated by persistent gastrointestinal issues due to acute GVHD, and TKI therapy was not reinstated. Diligent post-transplant monitoring is imperative regardless of post-transplant TKI therapy.

## Conclusions

In conclusion, we present the case of a patient initially treated with TKI for CML-CP, who subsequently progressed to MBP. The administration of a third-generation TKI as bridging therapy before allo-HSCT resulted in a sustained MR. Future studies may explore the potential for long-term outcomes in CML-MBP using third-generation TKIs both before and after allo-HSCT.

## References

[1] Shah NP, Bhatia R, Altman JK (2024). Chronic Myeloid Leukemia, Version 2.2024, NCCN Clinical Practice Guidelines in Oncology. J Natl Compr Canc Netw.

[2] Radujkovic A, Dietrich S, Blok HJ (2019). Allogeneic stem cell transplantation for blast crisis chronic myeloid leukemia in the era of tyrosine kinase inhibitors: a retrospective study by the EBMT chronic malignancies working party. Biol Blood Marrow Transplant.

[3] Shimazu Y, Murata M, Kondo T (2022). The new generation tyrosine kinase inhibitor improves the survival of chronic myeloid leukemia patients after allogeneic stem cell transplantation. Hematol Oncol.

[4] Hirose Y, Kiyoi H, Iwai M, Yokozawa T, Ito M, Naoe T (2002). Successful treatment with imatinib mesylate of a CML patient in megakaryoblastic crisis with severe fibrosis. Int J Hematol.

[5] Pelloso LA, Baiocchi OC, Chauffaille ML, Yamamoto M, Hungria VT, Bordin JO (2002). Megakaryocytic blast crisis as a first presentation of chronic myeloid leukemia. Eur J Haematol.

[6] Bryant BJ, Alperin JB, Elghetany MT (2007). Paraplegia as the presenting manifestation of extramedullary megakaryoblastic transformation of previously undiagnosed chronic myelogenous leukemia. Am J Hematol.

[7] Campiotti L, Grandi AM, Biotti MG, Ultori C, Solbiati F, Codari R, Venco A (2007). Megakaryocytic blast crisis as first presentation of chronic myeloid leukemia. Am J Hematol.

[8] Pullarkat ST, Vardiman JW, Slovak ML, Rao DS, Rao NP, Bedell V, Said JW (2008). Megakaryocytic blast crisis as a presenting manifestation of chronic myeloid leukemia. Leuk Res.

[9] Karkuzhali P, Shanthi V, Usha T (2013). A case of chronic myeloid leukaemia presenting as megakaryocytic blast crisis (AML M7). Ecancermedicalscience.

[10] Hino Y, Doki N, Yamamoto K (2016). [Chronic myeloid leukemia relapsing ten years after allogenic bone marrow transplantation]. Rinsho Ketsueki.

[11] Khemka R, Gupta M, Jena NK (2019). CML with megakaryocytic blast crisis: report of 3 cases. Pathol Oncol Res.

[12] Sasaki H, Mitani S, Kusumoto S (2019). Pre- and post-transplant ponatinib for a patient with acute megakaryoblastic blast phase chronic myeloid leukemia with T315I mutation who underwent allogeneic hematopoietic stem cell transplantation. Int J Hematol.

[13] Ureshino H, Shindo T, Sano H (2020). Reconstitution of NK cells expressing KIR3DL1 is associated with reduced NK cell activity and relapse of CML after allogeneic hematopoietic stem cell transplantation. Int J Hematol.

[14] Agrawal S, Kumar K, Singh M, Chandra H (2022). Megakaryocytic blast crisis in chronic myeloid leukiemia: an uncommon presentation in a common neoplasm. Hematol Transfus Cell Ther.

[15] Soverini S (2023). Resistance mutations in CML and how we approach them. Hematology Am Soc Hematol Educ Program.

[16] Copelan EA, Crilley PA, Szer J (2009). Late mortality and relapse following BuCy2 and HLA-identical sibling marrow transplantation for chronic myelogenous leukemia. Biol Blood Marrow Transplant.

[17] Goldman JM, Majhail NS, Klein JP (2010). Relapse and late mortality in 5-year survivors of myeloablative allogeneic hematopoietic cell transplantation for chronic myeloid leukemia in first chronic phase. J Clin Oncol.

[18] Carpenter PA, Snyder DS, Flowers ME (2007). Prophylactic administration of imatinib after hematopoietic cell transplantation for high-risk Philadelphia chromosome-positive leukemia. Blood.

[19] Olavarria E, Siddique S, Griffiths MJ (2007). Posttransplantation imatinib as a strategy to postpone the requirement for immunotherapy in patients undergoing reduced-intensity allografts for chronic myeloid leukemia. Blood.

[20] Carpenter PA, Johnston L, Fernandez HF (2017). Posttransplant feasibility study of nilotinib prophylaxis for high-risk Philadelphia chromosome positive leukemia. Blood.

